# ***Borrelia*** [bә-rel′e-ә]

**DOI:** 10.3201/eid1507.999999

**Published:** 2009-07

Named after French bacteriologist Amedée Borrel (1867–1936) in 1907, *Borrelia* is a genus of bacteria, family Spirochaetaceae, made up of gram-negative, irregularly coiled helical cells that surround a central fibrillar substance. These organisms cause tick-borne and louse-borne relapsing fever in humans and animals. For example, *B. hermsii,* transmitted by *Ornithodoros hermsi* ticks, causes relapsing fever in the Western United States, and *B. recurrentis* causes louse-borne relapsing fever worldwide. Another member of the genus, *B. burgdorferi,* isolated from patients with arthritis-like symptoms by Willy Burgdorfer and Alan G. Barbour in 1982, is the etiologic agent of Lyme disease.

Although Borrel did not work extensively with spirochetes, he published several articles on *Spirillum* (now *Borrelia*) *gallinarum*. He is also known for searching for an infectious cause of cancer and for proposing that this agent could be a virus.

